# Supplementary material for: A fault-intrusion-tolerant system and deadline-aware algorithm for scheduling scientific workflow in the cloud
Source: PeerJ Comput Sci. 2021 Nov 2;7:e747. doi: 10.7717/peerj-cs.747 (PMC8576554; doi:10.7717/peerj-cs.747)
Supplement: Supplemental Information 6 [file peerj-cs-07-747-s006.zip › Myproject1.1/docs/org/cloudbus/cloudsim/network/datacenter/RootSwitch.html]

RootSwitch


JavaScript is disabled on your browser.


- Overview
- Package
- Class
- Use
- Tree
- Deprecated
- Index
- Help

- Prev Class
- Next Class

- Frames
- No Frames

- All Classes

- Summary:
- Nested |
- Field |
- Constr |
- Method

- Detail:
- Field |
- Constr |
- Method


org.cloudbus.cloudsim.network.datacenter

## Class RootSwitch

- java.lang.Object
- - org.cloudbus.cloudsim.core.SimEntity
  - - org.cloudbus.cloudsim.network.datacenter.Switch
    - - org.cloudbus.cloudsim.network.datacenter.RootSwitch

- All Implemented Interfaces:
  :   java.lang.Cloneable

  ---

    

  ```
  public class RootSwitch
  extends Switch
  ```

  This class allows to simulate Root switch which connects Datacenter to external network. It
  interacts with other switches in order to exchange packets.
  Please refer to following publication for more details:
  Saurabh Kumar Garg and Rajkumar Buyya, NetworkCloudSim: Modelling Parallel Applications in Cloud
  Simulations, Proceedings of the 4th IEEE/ACM International Conference on Utility and Cloud
  Computing (UCC 2011, IEEE CS Press, USA), Melbourne, Australia, December 5-7, 2011.

  Since:
  :   CloudSim Toolkit 3.0

- - ### Field Summary

    - ### Fields inherited from class org.cloudbus.cloudsim.network.datacenter.Switch

      `BagofTaskVm, datacenterid, dc, downlinkbandwidth, downlinkswitches, downlinkswitchpktlist, fintimelistHost, fintimelistVM, hostlist, id, latency, level, numport, packetTohost, pktlist, switching_delay, uplinkbandwidth, uplinkswitches, uplinkswitchpktlist, Vmlist`
    - ### Fields inherited from class org.cloudbus.cloudsim.core.SimEntity

      `FINISHED, HOLDING, RUNNABLE, WAITING`
  - ### Constructor Summary

    Constructors

    Constructor and Description

    `RootSwitch(java.lang.String name,
    int level,
    NetworkDatacenter dc)`

    Constructor for Root Switch We have to specify switches that are connected to its downlink
    ports, and corresponding bandwidths
  - ### Method Summary

    Methods

    | Modifier and Type | Method and Description |
    | --- | --- |
    | `protected void` | `processpacket_up(SimEvent ev)` Send Packet to switch connected through a downlink port |

    - ### Methods inherited from class org.cloudbus.cloudsim.network.datacenter.Switch

      `getfreehostlist, getfreeVmlist, getHostwithVM, processEvent, processhostpacket, processpacket_down, processpacket, processpacketforward, shutdownEntity, startEntity`
    - ### Methods inherited from class org.cloudbus.cloudsim.core.SimEntity

      `cancelEvent, clone, getEventBuffer, getId, getName, getNextEvent, getNextEvent, getState, numEventsWaiting, numEventsWaiting, pause, run, schedule, schedule, schedule, schedule, scheduleFirst, scheduleFirst, scheduleFirst, scheduleFirst, scheduleFirstNow, scheduleFirstNow, scheduleFirstNow, scheduleFirstNow, scheduleNow, scheduleNow, scheduleNow, scheduleNow, selectEvent, send, send, send, send, sendNow, sendNow, sendNow, sendNow, setEventBuffer, setId, setState, waitForEvent`
    - ### Methods inherited from class java.lang.Object

      `equals, finalize, getClass, hashCode, notify, notifyAll, toString, wait, wait, wait`

- - ### Constructor Detail


    - #### RootSwitch

      ```
      public RootSwitch(java.lang.String name,
                int level,
                NetworkDatacenter dc)
      ```

      Constructor for Root Switch We have to specify switches that are connected to its downlink
      ports, and corresponding bandwidths

      Parameters:
      :   `name` - Name of the switch
      :   `level` - At which level switch is with respect to hosts.
      :   `dc` - Pointer to Datacenter
  - ### Method Detail


    - #### processpacket\_up

      ```
      protected void processpacket_up(SimEvent ev)
      ```

      Send Packet to switch connected through a downlink port

      **Overrides:**
      :   `processpacket_up` in class `Switch`

      Parameters:
      :   `ev` - Event/packet to process


- Overview
- Package
- Class
- Use
- Tree
- Deprecated
- Index
- Help

- Prev Class
- Next Class

- Frames
- No Frames

- All Classes

- Summary:
- Nested |
- Field |
- Constr |
- Method

- Detail:
- Field |
- Constr |
- Method
